# Modern Displacement Measuring Systems Used in Geotechnical Laboratories: Advantages and Disadvantages

**DOI:** 10.3390/s21124139

**Published:** 2021-06-16

**Authors:** Małgorzata Jastrzębska

**Affiliations:** Department of Geotechnics and Roads, Faculty of Civil Engineering, Silesian University of Technology, Akademicka 5, 44-100 Gliwice, Poland; malgorzata.jastrzebska@polsl.pl; Tel.: +48-32-237-1543

**Keywords:** geotechnical laboratory, soil parameters, strain measurement systems, X-ray tomography, stereophotogrametry, laser sensors, encoder sensors

## Abstract

The paper presents the contemporary displacement measurement systems used in geotechnical laboratories during the determination of soil precise mechanical parameters, e.g., the shear modules G: initial and in the range of small and very small strains. In the laboratory, researchers use standard sensors for measuring deformation, pressure, and force as well as modern measuring systems such as linear variable differential transformers (LVDT), proximity transducers (PT), magnetic encoder sensors with fiber Bragg grating (FBG), or methods based on laser or X-ray measurement. None of the measurements are universal and their use depends on the type of soil (cohesive, non-cohesive), its condition (loose or dense, stiff or very soft), and its characteristic properties (e.g., organic soil, swelling soil). This study points out the interesting equipment solutions and presents the guidelines for selecting appropriate methods of deformation measurement.

## 1. Introduction

The correct and precise determination of soil mechanical parameters is an essential element of geotechnical and engineering design. It is worth remembering that the end result, i.e., obtaining a specific parameter, consists of a sequence of activities, which includes appropriate sampling from the subsoil, their storage, transport, preparation for tests, selection of the research procedure (including appropriate equipment and measuring sensors), development and interpretation of results. In the case of soils, one universal method cannot be given. It should always be considered the type of soil (cohesive, non-cohesive), its condition (loose or dense, stiff or very soft), its characteristic properties (e.g., organic soil, swelling soil), and further use of the obtained parameters (construction type). Each of the steps mentioned is regulated by relevant standards. Their reliable implementation guarantees that the measurements realized during the tests, with the use of more or less advanced sensors, will allow the determination ofthe real values of parameters in a controlled and homogeneous state of stress and deformation.

The aim of this review is to draw attention to the potential of geotechnical laboratories in terms of equipping them with modern apparatus and systems for measuring sample deformations, as well as the possibility of modernizing standard devices. The issue arrangement and the scope of their presentation is the author’s own choice, related to his experience in geotechnical laboratory investigations and research passion. The author’s intention is to briefly elaborate the available deformation measurement systems, with a particular accent on those “older” (from the 1970s) in “newer” applications. At the same time, a lot of attention is paid to the latest, interesting solutions (in relation to strain measurement and results analysis) used in geotechnical laboratories. In this context, a series of guidelines in the form of “windows” based on various criteria, and presented in Section 7, may facilitate the selection of an appropriate strain measurement method.

## 2. Modern Measuring Systems

The gradual development of measurement methods is primarily related to technological progress, which results in newer sensors and methods of data recording. Regardless of whether the goal is to measure deformation, pressure, or force, there is a wide range of possibilities: from traditional mechanical devices (simple solutions such as springs, gears, tubes, etc.) to highly specialized, using induction phenomena (conductors, semiconductors), resistivity, photoelectricity (lasers), piezoelectricity (piezoelectric crystals) and other combinations of different phenomena. The reference point in a geotechnical laboratory is usually a triaxial apparatus, considered by most researchers to be the mother of other devices that use and adapt its modern solutions. Traditional measurements of the axial deformation of the samples tested in the triaxial apparatus, carried out outside the cell, introduce significant errors in the calculation of the deformations [1]. This is for two main reasons. First, the errors in specifying deformation of the sample are due to the susceptibility of the porous stones and the layers of lubricant (slip layer) that deform under increasing vertical load. Second, the errors occur due to imperfections in the smoothing and alignment of the sample surface in contact with the pedestal and top cap of the apparatus. It is not without significance that the sample tends to take a barrel shape, which causes a distinct inhomogeneity of deformation at the height of the sample. The measurement uncertainties are all the more significant, the smaller the sample deformation because the measurement errors are of a comparable size to the measured deformations.

**Figure 1:**
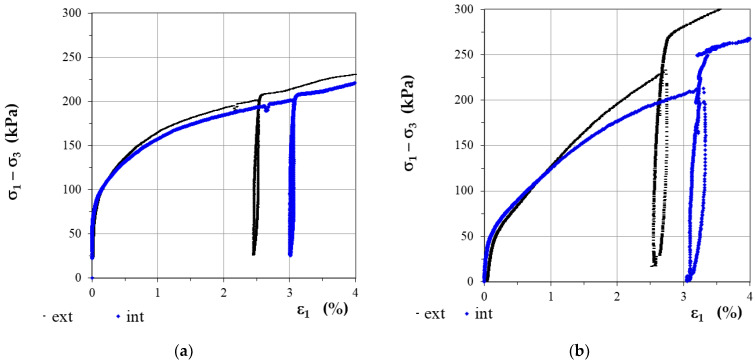
Comparison of the “stress-strain” characteristics in triaxial tests on clay for external (ext) and internal (int) measurement of deformation: (**a**) in undrained conditions; (**b**) in drained conditions; (after [2], via 2020 Creative Commons Attribution-NonCommercial-NoDerivatives 4.0 International Public License).

Due to the large inaccuracies of external measurements, various types of internal displacement measurement systems began to develop, allowing to directly measure the deformation of the sample itself in its central zone (omitting the disturbance zone at the point of contact of the sample with the base and the top cap). A comparison of the “stress deviator-axial strain” characteristics obtained using external and internal strain measurements is shown in Figure 1 [2].

### 2.1. Small Displacement Zone—Local Measurement

**Figure 2:**
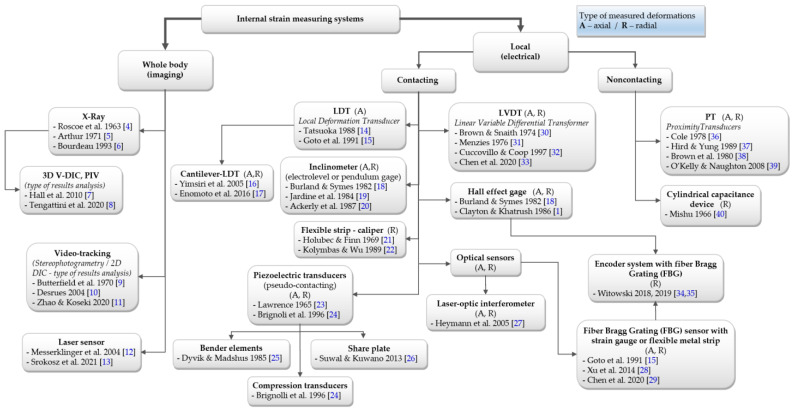
Division of internal deformation measurement systems (based on [3]; with permission via 2021 ASTM International License Number 5071920837322).

Figure 2 shows the general division of internal deformation measurement systems, based on [3], and referred to the inventors of the presented methods and the researchers who made a significant contribution to the next development of the technology [4,5,6,7,8,9,10,11,12,13,14,15,16,17,18,19,20,21,22,23,24,25,26,27,28,29,30,31,32,33,34,35,36,37,38,39,40].

**Figure 3:**
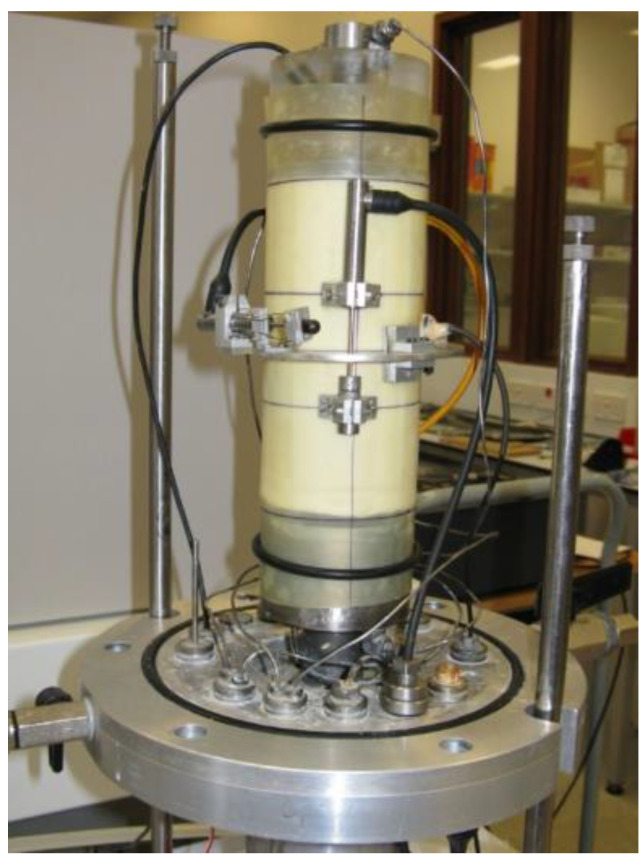
LVDT mounted on a sample (source: photo of Kowalska M., after [41]; available via 2021 Civil and Environmental Engineering’s permission).

**Figure 4:**
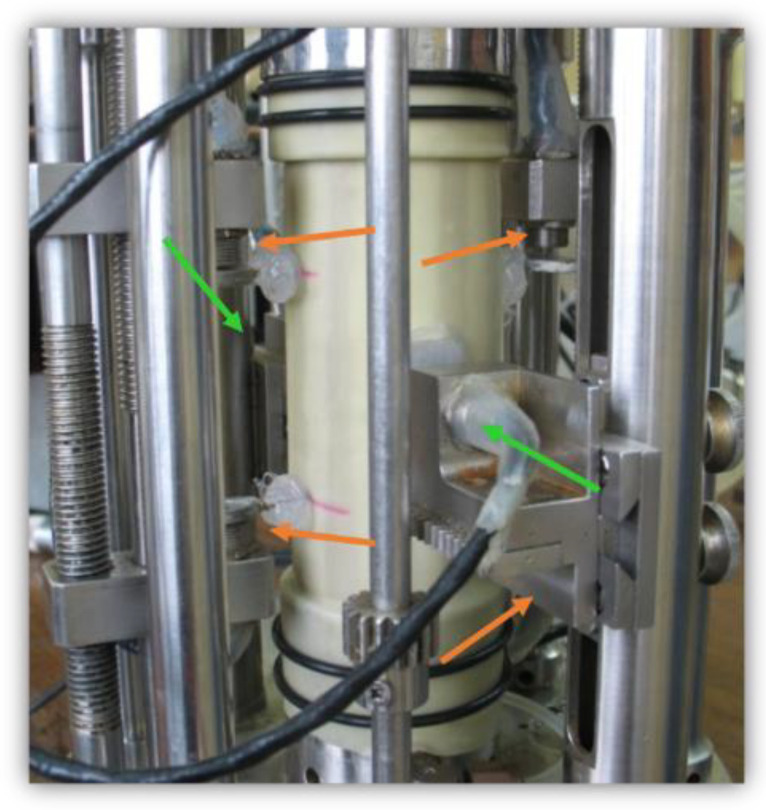
PT mounted on a sample (source: photo of Jastrzębska M., after [41]; available via 2021 Civil and Environmental Engineering’s permission).

There are two main groups of measurement systems: measurements covering the whole sample and local measurement, in which the sensors are inside the test cell. Local sensors may be attached to the sample (contact sensors, e.g., local deformation transducers (LDT), linear variable differential transformers (LVDT)), or not (non-contact sensors, e.g., proximity sensors (PT)). Figure 3 and Figure 4 show examples of measuring systems installed under real conditions.

All above-mentioned measuring systems, their advantages and limitations, were repeatedly reviewed and discussed, e.g., [42,43]. For this reason, as was mentioned earlier, the author of this study will not present the basic principles of system operation that have been known for a long time. The modifications of existing devices and new solutions conceptions are an essential objective of this review.

All soil measuring systems were originally dedicated to cylindrical samples in a triaxial apparatus. Due to the triaxial test condition, they should be resistant to long-term performance of varying pressure and temperature as well as to cell fluid type and high cell pressure. In addition, the type of soil (strong or weak), the sample dimensions (it is difficult to mount a certain system with sensors on a small sample), and the nature (axial or radial) and range of measured deformations are of great importance. It is also worth noting that the accurate measurement of radial deformation is more difficult than that of the axial one, as the radial boundary of a specimen which is in the rubber membrane, is less rigid and produces less uniform measurements.

Over time, these methods are modified and improved, especially those related to the measurement of radial deformations. For example, two new radial strain measurement devices were proposed by Chen et al. [29,33]. One of them is a system composed of two LVDTs mounted horizontally on a pair of yokes which are glued on the diametrically opposite sides of the specimen [33]. The second is the compass-type mechanism (so-called floating) composed of two metal legs connected by a hinge and two FBG sensors.

**Figure 5:**
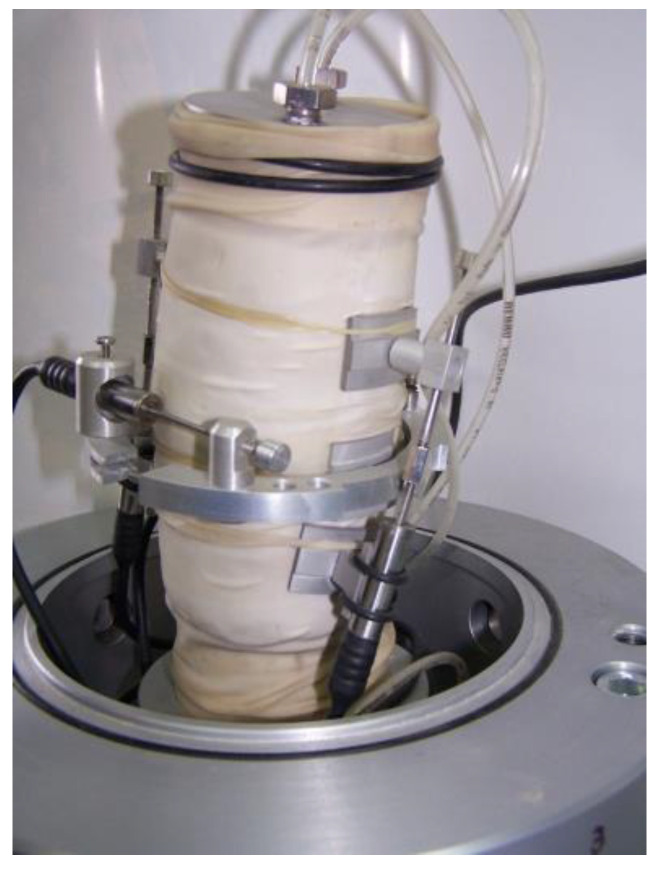
Sample of weak clay destroyed during the mounting of the sensors for strain measurement (source: photo of Jastrzębska M., after [41]; available via 2021 Civil and Environmental Engineering’s permission).

An added advantage is that the multiplexing capacity of FBG sensors enables the simultaneous measurement of strain or temperature at multipoints along one fiber line [29]. Chen et al. [29] admit that the proposed solution requires further research in the case of very soft clays. This doubt confirms this study author’s opinion that not all solutions are suitable for testing weak cohesive soils (too heavy mounting elements or too stiff cables) or highly compressible soils (too large deformations in the first phase of the loading sample). Figure 5 shows the example of a sample destroyed in this way [41].

**Figure 6:**
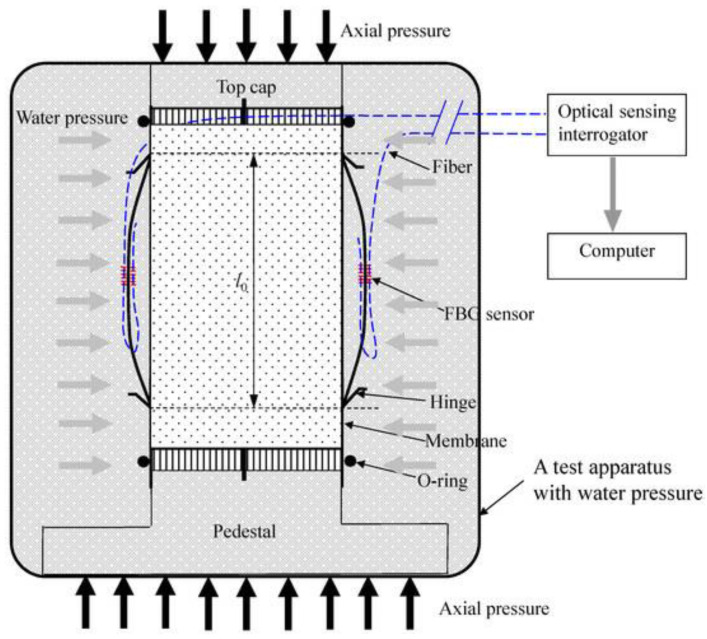
Schematic diagram of the SDTs on a soil specimen in a modified triaxial apparatus (after [44], available via 2009 license: CC BY 4.0).

Another type of small deformation transducer (SDT) based on fiber Bragg grating sensors was proposed by Xu (Figure 6, [44]). Xu showed that the SDT can be used for local deformation measurements (only the axial strains) of soil specimens in a modified triaxial apparatus as it has obvious merits such as light weight, high accuracy, resistance to corrosion and ease of handling [44].

**Figure 7:**
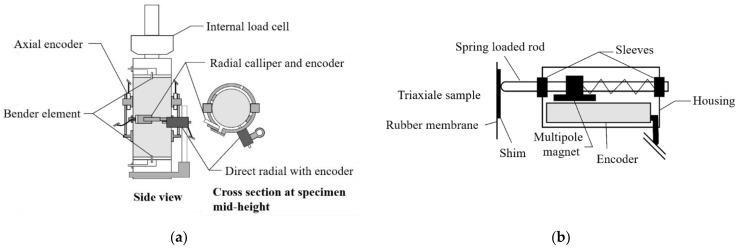
Magnetic encoders system with fiber Bragg grating (FBG): (**a**) small strain instrumentation; (**b**) cross section through direct radial transducer (after [35], available via 2019 license: CC BY 4.0).

The new magnetic encoders system with Fiber Bragg Grating (FBG) is another interesting proposition for researchers. This system is based on the Hall effect, with a wave receiver outside the triaxial cell, which eliminates the need for using cables [34,35]. The mounting elements from the LVDT system were using in this solution (Figure 7). This technical solution for measuring the axial and radial displacements of the cylindrical sample in triaxial apparatus has been covered by patent protection [45].

It can be seen that the popularity of the presented solutions also changes, and some, such as a flexible strip, radial strain caliper, or cylindrical capacitance device, are obsolete due to unsatisfactory measurement reliability or even difficulties in installation and the inability to apply them to all soil types.

### 2.2. Necessity of Strain Measuring—Modules G, E, K and Poisson’s Ratio

**Figure 8:**
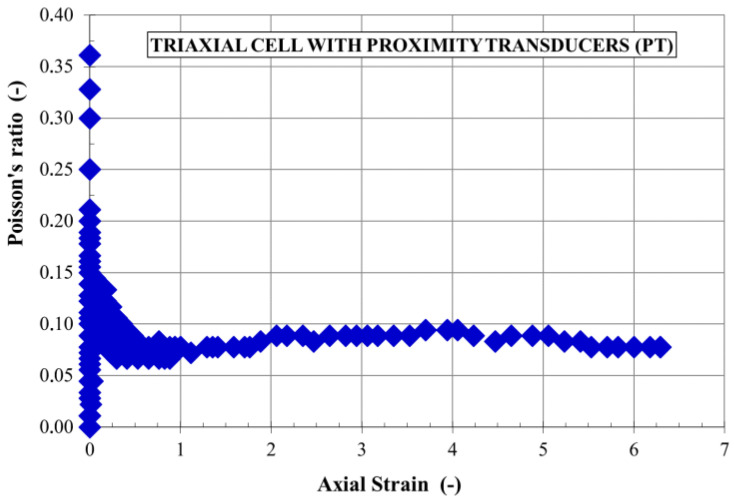
Variation of Poisson’s ratio of clay [48].

**Figure 9:**
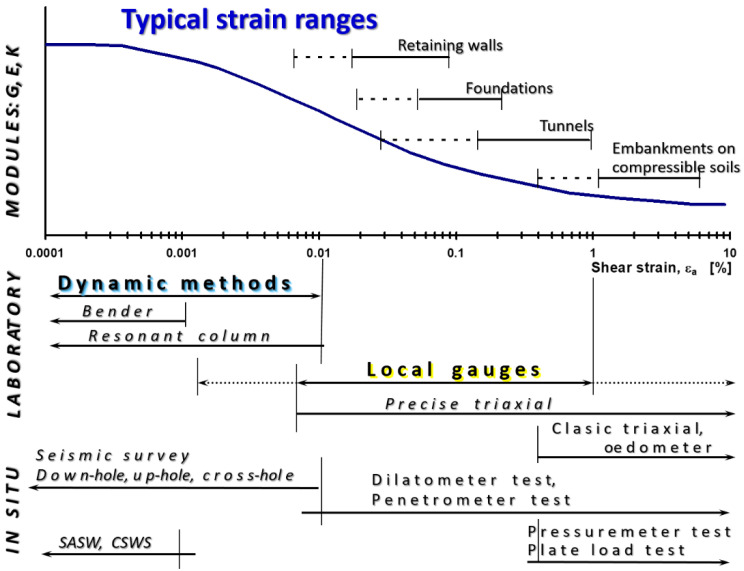
Approximate strain ranges corresponding to various measuring techniques of soil stiffness characteristics in laboratory and field tests, with the strain range marked by the typical geotechnical construction work (on the basis of [49,50,51,52]).

The issues related to very small and small deformations (of the order of 10^−3^ and less) date to the beginning of the 1970s, when extensive research was undertaken on the properties of soil response to dynamic loads [46]. The conclusion from these experiments was surprising. It turned out that the commonly known modules: “dynamic” (determined in a resonance column) and “static” (determined during the triaxial test), are one, and the same shear modulus (secant or tangential), dependent on the shear strain [47]. These modules are not two different elastic constants. The following ranges of deformations [20] have been experimentally distinguished: very small deformation (<10^−5^), small deformation (10^−5^–10^−2^) and large deformation (>0.01), in which the modulus values (shear modulus, G, modulus of elasticity, E, and bulk modulus, K) are different. A similar situation exists in the case of Poisson’s ratio, which is also not characterized by a constant value, as many engineers believe (Figure 8) [48]. Each deformation area corresponds to a different apparatus and the method of strain measurement (Figure 9, based on [49,50,51,52]). The importance of the small and very small strain zone is proved by the fact that the deformations that occur in the subsoil for most geotechnical design issues, e.g., retaining walls, underground structures (tunnels), and large-area foundations, are contained in the range of small deformations. Including the modules at large deformation in the geotechnical calculations leads to unnecessary oversizing of the structure [53].

This has been experimentally confirmed many times, e.g., [1,15,19,25,49,54,55,56,57,58,59,60,61,62,63]. To obtain the full characteristics of modulus variability (from very small to large deformations), it is necessary to properly select the test methods, all results combined of which will cover the full range of deformations. There is no one universal test (neither field nor laboratory) that would allow obtaining results from the entire range of deformations. Figure 9 shows the measurement accuracy ranges of individual test methods, both laboratory and field, and indicates the range of deformations corresponding to the operation of typical geotechnical structures.

**Figure 10:**
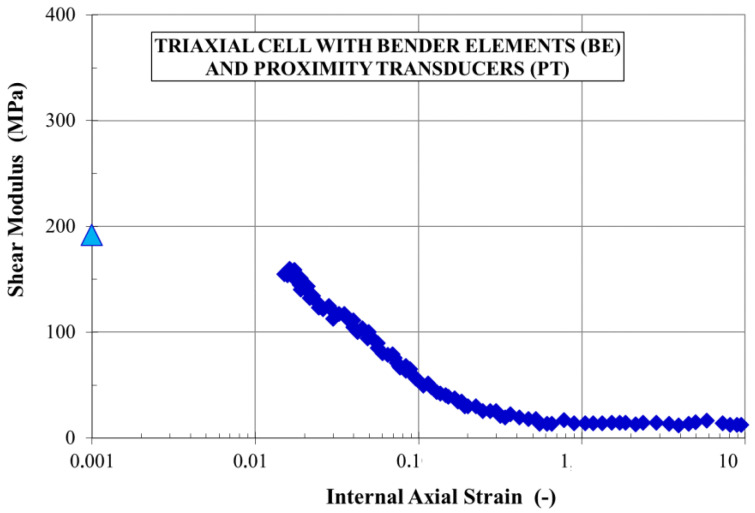
Maximum values of shear modulus G obtained from tests with wave velocity measurement and the rest obtained from tests in a precise triaxial apparatus with an internal measurement system [62].

As a result of laboratory tests, the full variability of strain modules (G, E, K) can be obtained with a minimum test set, which is a precise triaxial apparatus with local strain measurement (contact or noncontact sensors) and the bender elements (BE) mounted in its pedestal and cap. In this way, it is possible to resign from complicated tests in the resonance column. This is a good solution because, as demonstrated by Kuwano and Katagiri [64] on the basis of a questionnaire conducted among 92 geotechnical laboratories, only 20% of laboratories declare a resonant column, while barely 3% confirm their use several times a year. In the final analysis, the missing part of the graph can be adjusted knowing its initial value (G_max_, E_max_, K_max_ from acoustic wave measurement-bender elements) and its further part based on the local measurement of deformations as shown in Figure 10.

It is worth mentioning that originally, the BE system was installed in a triaxial apparatus. The fact is that the bender elements can be mounted in any geotechnical device capable of controlling stresses while measuring deformations, or vice versa, as mentioned by Ferreira [42]. Such equipment includes: odometer [65,66,67,68,69,70,71,72], direct shear apparatus [65,73], resonant-column [74,75,76,77], centrifuge [78,79], hollow cylinder [80,81,82], calibration chambers [83,84], true triaxial and cubical cel apparatus [85,86]. Some examples are presented in Section 6.

It is worth noting that other types of piezoelectric transducers are also used in geotechnical laboratories. These are a shear-plate (SP) and a compression transducer (CT). A shear-plate consists of a single piezoceramic element and its first use was reported by Lawrence [23,87], next by Brignoli et al. [24], and Ismail and Rammah [88]. Brignoli et al. [24] confirmed that for undisturbed stiff soils and coarse-grained soils, shear plates are better suited than bender elements since (SP) do not penetrate into the soil. In recent years, the intensive experiences have been carried out on using share plates as an alternative to bender elements for granular soil testing [26,89].

A compression transducer, similarly to a shear-plate, consists of a single piezoceramic element. It was only used in the ISMES-Enel.Hydro triaxial apparatus [24] and it is rather recommended as a complement to bender elements or share plates. More on the emerging new technology and the role of piezoelectric transducers in tests for a number of soil mechanical properties (such as creep, fracture toughness, hardness, and impact toughness) can be found in the Mohammed et al.’s state-of-the-art [90].

## 3. Localisation and Development of Deformations in the Sample

Another group of experiments is related to the need for increasingly accurate registration of the localisation of deformations and the analysis of their development in a sample (material) under load, not only in geotechnics, but in the entire construction engineering. This issue is closely related to technological development and new opportunities. During several decades, new methods of recording displacements have appeared, based on optical (graphic) measurements, measurements using X-ray, thermal, electromagnetic, and other radiation. These methods are called “full-field methods” in experimental mechanics. Although they are included in the so-called internal systems, it should be remembered that all devices (video cameras, laser sensors, tomographic scanners) taking readings of deformation changes are located outside the test cell, not inside.

### 3.1. Nature of Deformation

Referring to the foregoing, the simplest method of recording the location of deformations and, at the same time, the starting point for other methods are observations made directly in the field. The effects of certain visible phenomena may sometimes surprise to their range. As is known, earthquakes and landslides cause visible large-scale deformation of the terrain. Numerous field observations of the deformation effects in soil and rocks have been reflected in small-scale studies on samples in the laboratory. The aim of the laboratory experiments is not only to observe the deformation after shearing of the sample, but also to observe and analyze its evolution during the test.

Currently, a dynamic development of the laboratory observation methods of the deformation location in the soil can be observed. Thanks to these methods, it is possible to locate the deformations (in the form of shear bands or fractures) appearing in the sample during shearing increasingly precisely and to analyze the soil structure changing. Similar methods are used in the case of rocks, but in this study, the methods used for soil testing will be presented in the following part.

### 3.2. Overview of the Available Noninvasive Measurement Methods

In a very wide range of applications, non-invasive methods of observing the full field of deformations and displacements include the following methods (based on [91,92]:

- optical,
- based on X-rays (X-ray tomography), e.g., [7,93,94,95,96],
- neutron tomography, e.g., [8,97,98],
- thermography,
- acoustic and ultrasonic tomography, e.g., [99,100,101],
- Magnetic resonance imaging (MRI), e.g., [102],
- Electrical resistivity tomography (ERT), e.g., [103],
- Positron emission tomography (PET).

The methods most often used in construction engineering and in geotechnical laboratories include (often combining them), optical methods and methods using X-rays.

Optical methods include (based on [91,92]):

1. pictures:
  - observation of the reference points (so-called markers) and the deformation of the grid with equal meshes,
  - photogrammetry/stereophotogrammetry,
  - Digital image correlation (DIC).
2. mesh method/Moiré effect analysis,
3. interferometry:
  - Moiré interferometry,
  - Electronic (Digital) speckle pattern interferometry (ESPI) or (DSPI),
  - holographic interferometry or optical holography.

It is worth paying attention to the fact that it is difficult to make a strict division into individual methods, because in most cases the principle of their operation is very similar and does not differ too much. It is also problematic to state unequivocally what is an “observational method” and what is a “measurement method”. Often times, these methods overlap and complement each other; for example, X-ray test would not make sense if there were not digital image correlation methods. Similarly, the stereophotogrammetry is based on the observation of grid deformation and appropriate processing of recorded digital images.

The methods used to analyze the obtained digital images include:

- DIC—digital image correlation,
- V-DIC or DVC—volume digital image correlation or digital volume correlation,
- PIV or PTV—particle image velocimetry or particle tracking velocimetry.

Although the PIV method was originally derived from fluid mechanics, today it is very often equated with the DIC method. Both methods use correlation techniques based on the light (and gray) distribution and on the tracing of the particle distribution pattern in the base image and the subsequent images. The use of the PIV (DIC) method is widespread. First of all, it is used to identify the strain localization in loose materials. For example, the PIV (DIC) method is used during tests in various geotechnical devices (e.g., biaxial apparatus-element tests; [10,104] and in modeling the behavior of granular soil behind a retaining wall (model test; [105]). By combining the PIV method with digital photography and the discrete element method (DEM), it is possible to analyze granular media on the microstructure (single grain) level.

Looking for correlation between standard strain measurements with LVDT sensors and digital image correlation methods, Hosseini et al. [106] showed the good agreement of the results obtained with both techniques. Given the low cost and the ability to determine the full-field displacement, they considered PIV (DIC) a good alternative to conventional measurement techniques. This was confirmed, inter alia, by Srokosz et al. [13] in non-cohesive soil research in a resonant column using local proximity sensors and LVDT sensors. Soil specimens’ deformations (in the small strain range) during the torsional shearing (TS) test are examined by using particle image velocimetry (PIV) according to the SIFT (scale-invariant feature transform) optical flow code developed in the MATLAB environment by Liu [107].

The significant development of digital technology, combined with the ease of analyzing digital images, has also caused the replacement of analog photography with digital photos, especially since the principle of measurement is the same: displacements and deformations are determined by overlapping consecutive images. The digital method is fully automated, and the amount of data processed is incomparably greater. On the other hand, full automation of the process carries the risk of anomalies in the obtained results, which are very sensitive to all mathematical assumptions in the algorithm. In addition, the distortions in the received digital images can be expected. Due to the fact that the photographed sample is inside the rubber membrane, which deforms with it, but optically “smoothes out” the deformation areas, the soil shear band which is a few grains wide looks much wider in the obtained images than in reality.

Despite some disadvantages and imperfections of this method, it is a tool that definitely improves and facilitates the observation of developing full-field deformations in geomaterials. For this reason, digital technology has almost replaced the traditional analog technology.

### 3.3. Stereophotogrammetry—2D Optical Methods to Study Strain Localisation in Soil

Stereophotogrammetry, which is a type of photogrammetry, is a technology that allows to reproduce the shape, sizes, and mutual position of objects in three-dimensional space on the basis of a photo pair (stereograms). Most often, photogrammetry is associated with aerial photogrammetry (a wide coverage area), but it is also used for special purposes, e.g., to study full-field deformation of the sample in geomechanics.

In essence, there are two types of stereograms that give the so-called (based on [10]):

- False image—the photographs taken from a fixed viewpoint at different times during the loading process (false relief stereophotogrammetry (FRS)),
- Real image—the photographs taken from different points in space at the same time.

**Figure 11:**
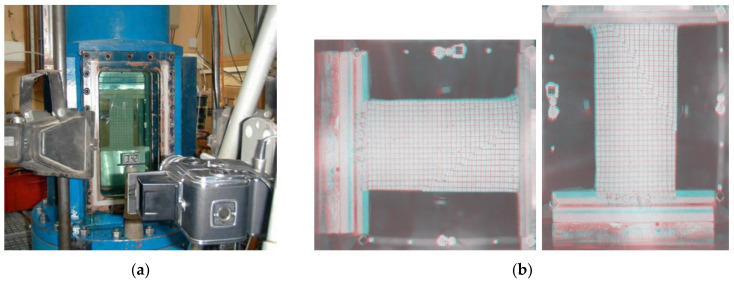
Test stand with the use of stereophotogrammetry in Laboratory S3R: (**a**) biaxial apparatus with two-camera system (source: phot. of Jacques Desrues, 1999); (**b**) camera record at the moment of failure (source: phot. of Leonardo Lenti, 2000); (after [109], available via 2021 Desrues’ permission).

The first application of stereophotogrammetry in soil mechanics was in 1970 by Butterfield et al. [9]. Since then, along with technological development, this method has only been improved. In the geotechnical laboratory, stereophotography uses optical devices to measure the full-field displacement of a sample under loading. It can be, for example, a system of two cameras placed on both sides of the sample (Figure 11a), which at the same time record changes in two different planes. An example of such images is shown in Figure 11b. The sample has a system of reference points (in the form of a grid applied to the sample—as, e.g., in Figure 11), which make it possible to recreated the external orientation of the images.

**Figure 12:**
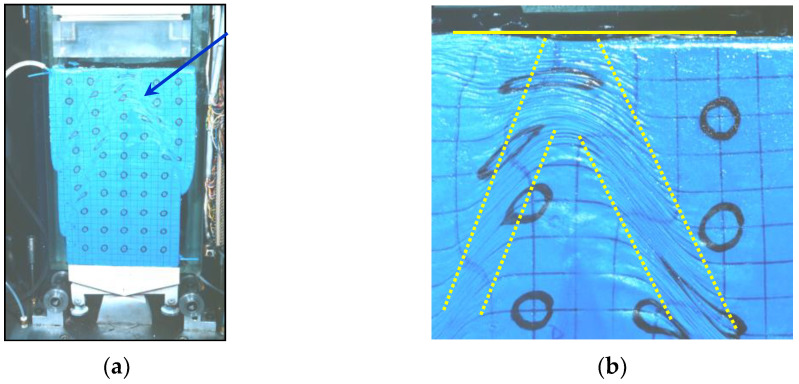
Observations made during the biaxial test: (**a**) general view of the biaxial apparatus with a sample after the test; (**b**) multiple shear bands of nonzero thickness, wide deformations, and «reflection» on the upper surface (source: phot. of Jacques Desrues, 2003; (after [109], available via 2021 Desrues’ permission).

The deformations observed in the sample will be seen as convex (Figure 12). This method is not perfect. It suggests that all sample deformations occurred simultaneously.

**Figure 13:**
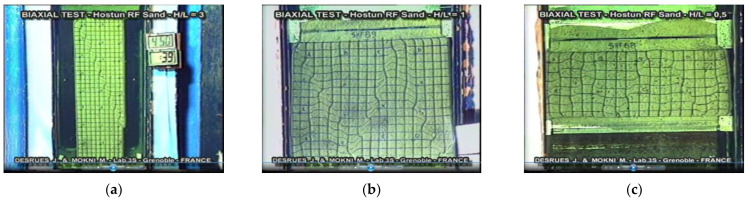
The influence of Hostun RF sand sample dimensions in the biaxial compression test on the bifurcation phenomenon and the range of disturbance zone, on the example of images recorded by stereophotogrammetry: (**a**) sample of dimensions H/D = 3; (**b**) sample of dimensions H/D = 1; (**c**) sample of dimensions H/D = 0,5; (source: video film of Desrues J. and Mokni M. 2000; based on [109], available via 2021 Desrues’ permission).

On the other hand, the advantages of stereophotogrammetry include the registration of all areas of deformation, even those that may disappear during the test. Stereophotogrammetry also helps to assess the influence of bending conditions and sample slenderness on the formation of various shear bands (bifurcation phenomenon, e.g., [108], Figure 13): parallel, crossing and temporary deformation areas.

Currently, researchers are using photogrammetry in a triaxial tests [110,111,112] and in torsional shear tests on a hollow cylindrical specimen [11]. The cylindrical shape of the test cell and the sample in the triaxial apparatus require correcting the algorithms for data analysis and taking countermeasures (especially in the case of shear torsion). Such actions are especially necessary in the case of torsional shear due to a continuously changing geometric relationship between the tracked target on the specimen surface and the camera lens [11]. The proposed modifications include, i.a., the use of a plane-shaped cell on the side facing the camera, the reflection mirrors next to the specimen in the triaxial cell [113], the use of three digital cameras during the test [11].

## 4. RTX-Based Methods

The first application of X-ray tomography in soil mechanics was in the early 1960s in Cambridge as a noninvasive technique for measuring strain field in soil which was documented by the Roscoe’s group [4] and later by Arthur [5]. From the early 1980s, this method was developed intensively by the researchers’ the group from Laboratory 3S-R in Grenoble (e.g., [10,94,95,96]) and later by Alshibli et al. [114]. The radiography measurement is carried out in the horizontal plane from different angular positions around the sample. On the basis of the intensity of the X rays, it is possible to determine the density distribution inside the sample, which is not possible with other methods, e.g., using stereophotogrammetry. A certain type of observed deformation (e.g., isochoric deformation without volume change, and thus invisible in tomography images) was forced to complement the X-ray tomography with 3D-volumetric digital image correlation (V-DIC) at the stage of final analysis [94].

Generally, there are three types of scanners for X-ray tomography (XRT): medical scanners, industrial scanner, and synchrotron. These devices differ in the method of generating X-rays and the time of measurement.

### 4.1. Use X-rays in the Triaxial Tests—Tomotriax Apparatus

**Figure 14:**
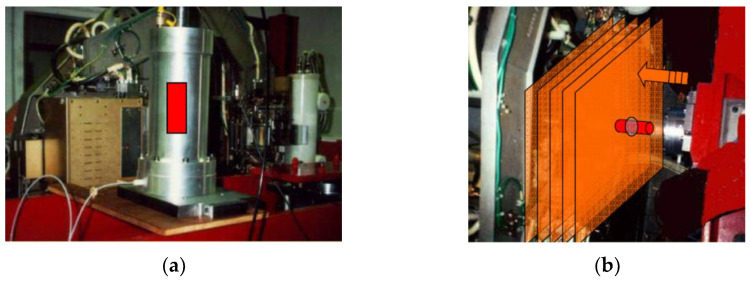
Tomotriax: (**a**) triaxial cell with selected sample, (**b**) triaxial cell placed in CT ready for operation in the scanner; (source: photo of Desrues J. from 3S-R Grenoble and LMA Marseille, 2000; after [109], available via 2021 Desrues’ permission).

One example of the use of X-rays in a geotechnical laboratory is the combination of a triaxial apparatus with a scanner. With such a device, called TOMOTRIAX (Figure 14), Desrues and coworkers from a Laboratory: 3SR in Grenoble and LMA in Marseille could carry out the experimental investigations of strain localization in sands.

Using X-ray tomography in a quantitative way requires a calibration of CT values to obtain the density distribution or porosity index inside the sample [92]. It is an essential soil parameter, because most often the greatest deformations are observed in zones of lower density (samples with heterogeneous density). In addition, knowing the void ratio of the shear plane provides information about its limit value at which failure occurs, and which is independent of the initial condition of the sample (a constant for a specific soil).

### 4.2. Use X-rays in Triaxial Tests—A Synchrotron

#### 4.2.1. Characteristics of the Synchrotron

The next stage of technological development in full-field analysis is the use of the synchrotron in soil research as a device combining three basic advantages: generation of much stronger X-rays, more precision and scanning speed. Higher energy and stronger photon beams result in higher image resolution, even to the micrometric scale. Such accuracy may not be as important in the case of coarsegrained soils, such as sand, for which the width of the shear band is about 10–20 grain diameters (about a few millimeters). However, it is important during the test deformations in finegrained soils, e.g., clays, in which grains are much smaller, and thus the shear bands are definitely narrower. Now, there are around 40 large synchrotron light sources over the world, in about 20 countries. The largest synchrotron in the world is the LHC (Large Hadron Collider) in Switzerland (energy—7000 GeV, circumference—about 27,000 m). The synchrotron with the widest energy spectrum from microwaves to gamma rays and the first in the world with high-energy source, is the ESRF-EBS (European Synchrotron Radiation Facility—Extremely Brilliant Source) in Grenoble (energy—6 GeV, circumference—844 m).

#### 4.2.2. European Synchrotron Radiation Facility—Extremely Brilliant Source (ESRF-EBS) in Grenoble

**Figure 15:**
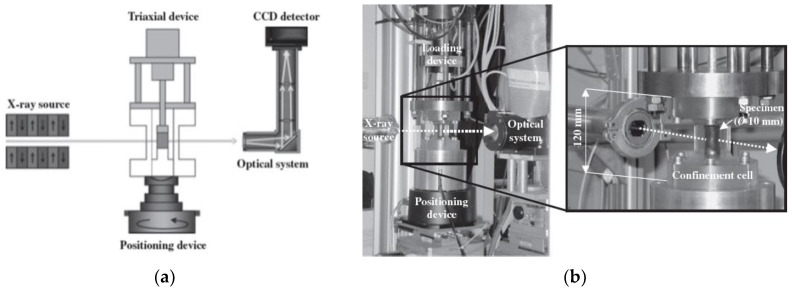
Schematic diagram of the research system in ESRF-EBS synchrotron: (**a**) scheme of the micro-CT device for triaxiel testing; (**b**) experimental setup showing a specimen inside the transparent triaxial cell; (based on [94], with 2021 permission of John Wiley and Sons, License No 5063850124930).

One of the most important European synchrotrons is the European Synchrotron Radiation Facility (ESRF), from 2020, called ESRF-EBS, after ESRF facility upgrade. It became the first fourth-generation high-energy synchrotron in the world. The facility is equipped with 45 experimental beamlines and the triaxial soil tests in-situ are carried out in one of them. In this particular case, “in-situ” means that X-ray tomography and image recording occur at the same time as loading the sample, without stopping the press while scanning. A diagram of such a system is shown in Figure 15a. Since 2003, the following three test stands (called Microtomotriax) are provided on the ID15 test line (ESRF-EBS), for triaxial tests on samples from rocks and cohesionless soils (Figure 15b):

1. Microtomotriax 1:
  - UU type test (unconsolidated and undrained shearing), max. confinning pressure in the cell σ_3_ = 1 MPa, sample dimensions: diameter φ = 20 mm, height H = 40 mm;
2. Microtomotriax 2:
  - CD or CU type test (consolidated and drained shearing or consolidated and undrained shearing)), σ_3_ = 1 MPa, sample dimensions: φ = 20 mm, H = 40 mm;
3. Microtomotriax 3:
  - UU type test (unconsolidated and undrained shearing), σ_3_ = 10 MPa, sample dimensions: φ = 10 mm, H = 10 mm.

#### 4.2.3. Analysis of the Results—Combined X-ray Tomography and 3D DIC (V-DIC)

The described X-ray tomography technology opens up new possibilities for understanding soil mechanics (in three-dimensional space), due to its high resolution and thus the possibility of analyzing the kinematics of single soil grains and interactions between all grains in the entire sample volume, during the loading process [92,93,94]. This method is not perfect, because in some cases it is difficult to trace the shear plane which is only visible if they are high compaction or loosening (dilatancy or crack opening) in its zone. Such a limitation can be overcome by complementing X-ray computed tomography with 3D digital image correlation (DIC), which is a mathematical tool to define the best mapping of an image into another. As a result, small deformations invisible in the grayscale, will be well observed. Additionally, using an appropriate technique, it is possible to “view” individual grains in the sample at their actual sizes. As a result of using the continuum-volume digital image correlation (CV-DIC), successive images of vertical slices of the sample are obtained during the triaxial loading. Based on the weakening of rays in the subsequent images, maps of porosity and axial deformation are generated. It is also possible to obtain an image of the grain rotation in the sample with respect to its own axis or the center of the sample, but then another method of image processing is used, namely, the discrete volume digital image correlation (DV-DIC).

X-ray images can be binarized to the grain and pore scale and split to identify and label individual grains so that changes in their position can be recorded. The color of the grains depends on the amount of rotation or displacement, while grains that are not “tracked” are left blank so as not to obscure the image.

## 5. Laser Methods

Although each of the optical methods (video cameras, laser sensors, tomographic scanners) has been developed for many years (laser sensors are relatively the youngest; the first mention of them appeared in 2004 (e.g., [12,115]), their most serious disadvantage —the optical distortions of the sample recorded image, is due by:

1. an image recording by the glass or plexiglass cell wall (inhomogeneity of the material) and by a liquid in the cell,
2. an influence of the test cell curvature,
3. an influence of the correct selection of sample lighting.

Taking into account the curvature of the test cell, its shape was changed from cylindrical to cuboid, thanks to which the optical image distortions have been reduced. This and other improvements (e.g., using contrasting colors of the grid with marks on the sample and illumination with an LED lamp) have reduced the optical image distortions. They have been used successfully in research in the studies of biaxial compression using stereophotogrammetry (Section 3.2) and in triaxial test by using laser measurement of deformation [12,16].

Messerklinger et al. [115] modified Romero et al.’s triaxial apparatus [116] in-house at the Institute for Geotechnical Engineering in Zurich. They installed three lasers around the sample, spaced at 120°, to obtain radial and volume displacement measurements of the sample. The axial displacements were measured inside the cell by the external LVDT sensor, which was mounted above the top cap, but below the load cell. Messerklinger et al. [12], such as Srokosz et al. [13], investigated the effect of measuring radial deformations with the use of internal LVDT sensors and external laser sensors on the characteristics of soil stiffness (shear modulus G) and the determination of the initial sample volume. It was seen that radial strain measurement with the laser scanning the device turned out to be more precise in the range of small and very small strain.

## 6. Interesting Solutions

### 6.1. Odometer Test—An Odometer Capable of Measuring Lateral Stresses

In odometric tests, cylindrical soil samples, placed in a rigid and non-deformable ring, are subjected to increasing vertical stress. The measured settlement is the response to this load. Generally, the results analysis does not take into account the horizontal component of stress, which is necessary to determine an initial state of the granular soil (contractive or dilative) defined by a point relative to the steady-state line in the stress space.

Świdziński [117,118] developed this conception thanks to the experiments carried out in a modernized odometer in the geotechnical laboratory in IBW PAN (Institute of Hydro-Engineering, Polish Academy of Sciences) in Gdańsk. The lateral stress was measured indirectly with a strain gauge installed on the cylindrical wall of the odometer. To this purpose, a small fragment in the middle was cut to a thickness of about 1 mm (standard wall thickness is about 6 mm) on the outer wall of the odometer ring. A tensometer measuring local horizontal deformation was placed inside the received cavity.

### 6.2. Odometer Test—A Miniaturized Odometer with an Optical Microscope Function

**Figure 16:**
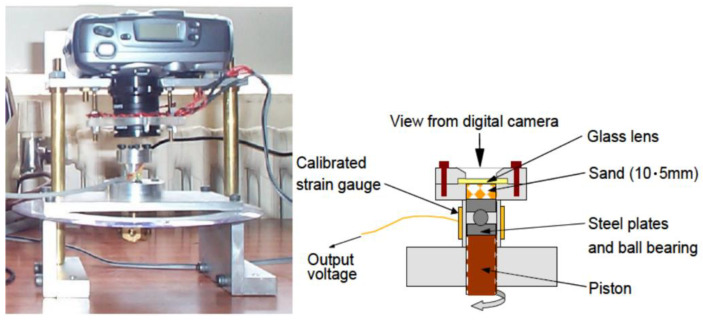
Set-up of miniature odometer for capturing images of microstructure during 1D compression (based on Figure 4 from [119]; reproduced by 2021 permission of Taylor & Francis Group).

Bolton and his co-workers [119] at the University of Cambridge conducted grain-scale and macroscopic observations in the element and model tests. This field of interest is called “clastic mechanics”. They use standard digital photography combined with particle image velocimetry (PIV) to analyse the phenomenon of soil grain fracture and its relationship with compressibility. For this purpose, they proposed the so-called miniaturized odometer (Figure 16) 10 mm in diameter, which was designed to observe using a digital camera CCD (charge coupled device) the breakage of 5-mm-high samples of dry sand behind a glass lens during one-dimensional compression.

### 6.3. Bender Elements—Non-Standard Installation in an Odometer and a Direct Shear Apparatus

**Figure 17:**
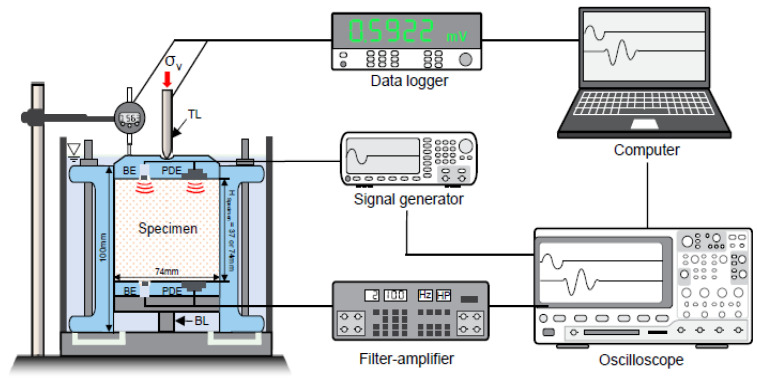
Schematic drawing of a modified odometric cell with electronics. TL and BL denote the load cells installed at the top and bottom plates, respectively. In addition, BE and PDE denote the bender elements and piezo-disk elements (after [71], in accordance with 2021 Creative Common CC BY License of MDPI).

Another interesting solution was proposed by Lee et al. [71] from Korea universities, who evaluated the effect of side friction (in conditions of full or incomplete sample saturation) on the stress dependence of the elastic wave velocities on the top and bottom of the silica sand sample in an odometer. For this purpose, they were modified an odometer cell by installing a pair of piezo disk elements and a pair of bender elements at both top and bottom plates (Figure 17).

**Figure 18:**
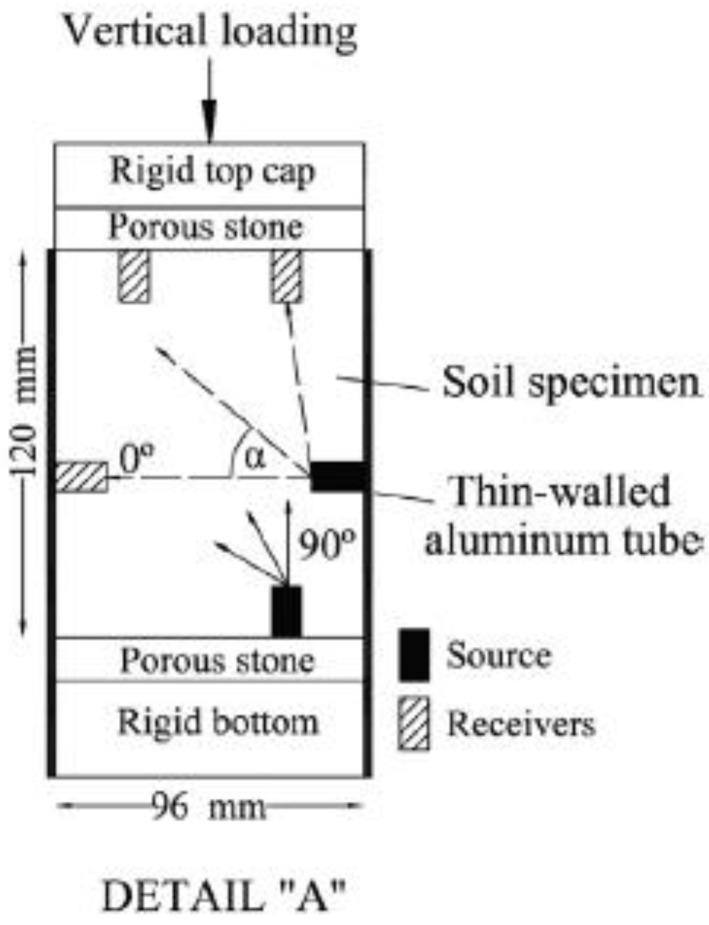
Schematic peripheral equipment and arrangement of bender elements [72] (available via 2020 license CC BY-NC-ND 4.0).

In turn, Chamorro-Zurita and Ovando-Shelley [72] from the National Autonomous University of Mexico (UNAM) were investigating the lacustrine soils and an anisotropy of their shear stiffness modulus at very small strain levels under static and dynamic conditions. They constructed a large-size odometer apparatus (sample size: diameter—96 mm, height—120 mm) made of thin-walled aluminum tubes with drilled mini two holes to accommodate the lateral bender elements in the sample (Figure 18). Thanks to this research, Chamorro-Zurita and Ovando-Shelley [72] confirmed the correlation between the yield stress (or overconsolidation ratio, OCR), the anisotropy ratio, the type of anisotropy (inherent or induced anisotropy) and the liquidity index (I_L_).

**Figure 19:**
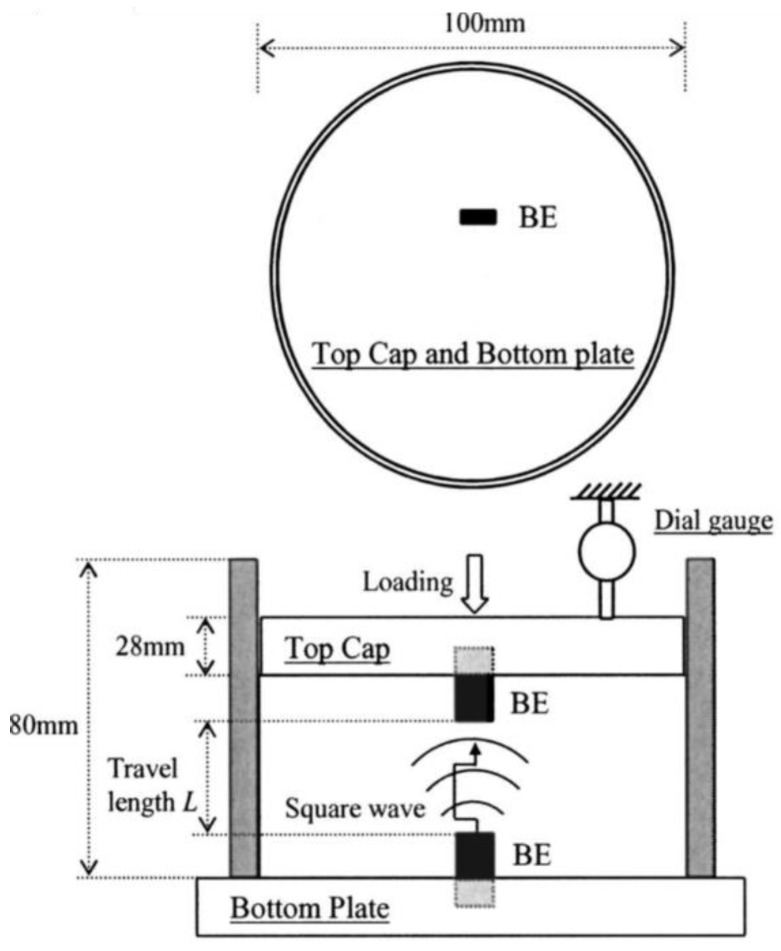
Odometer cell where bender elements are mounted in top cap and bottom plate (after [69], with permission from 2021 ASCE).

**Figure 20:**
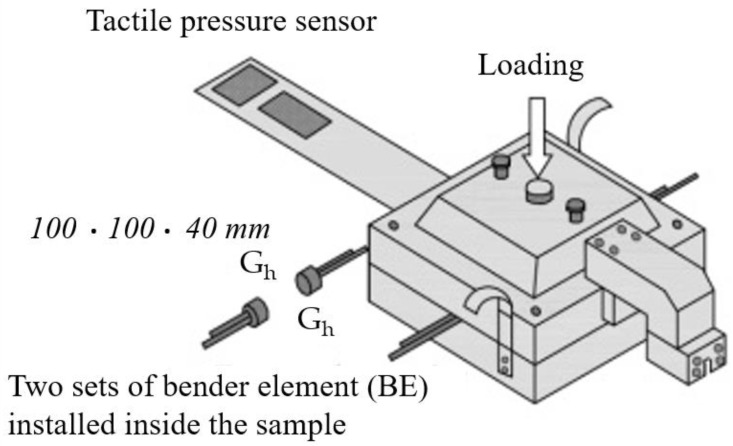
Tailor-made odometer with the tactile pressure sensor and two sets of bender elements installed inside the sample (after [70]; available via 2013 ISSMGE’s permission concerning the conference materials).

It is worth mentioning, that the first studies on a self-modernized odometer with installed the bender elements were performed by Yun and Santamarina [69] (Figure 19). They considered the small-strain stiffness of lightly cemented sandy soils. Later, Wang et al. [70] from The Hong Kong University of Science and Technology were investigated (in the macro-scale), the engineering soils properties originate from particle interactions. The tactile pressure sensor (film-like sensor) to monitor the evolution of contact normal forces among particles in aged sand and the bender elements were installed in a tailor-made odometer (Figure 20).

However, the earliest mentions of the use of the BE systems in odometric tests come from 1985 and a little later [65,66,67].

**Figure 21:**
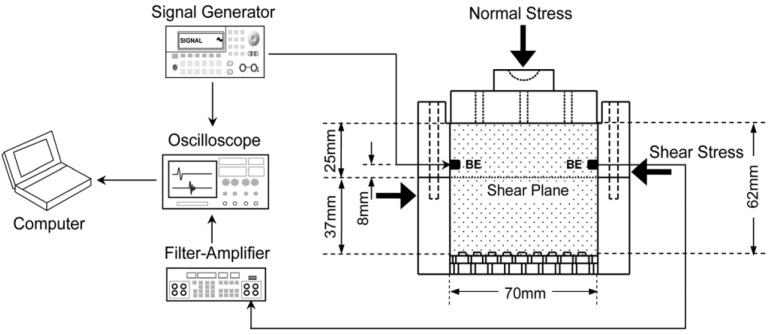
Direct shear box and peripheral electronics. BE denotes bender elements (after [73], with permisson from 2021 ASTM International—License Number 5062700491354).

Another group of researchers, Byun et al. [73] improved the classical direct shear apparatus by bender elements (Figure 21). In this way, they were investigating the shear strength and stiffness characteristics (at small strain) of the hydrophobic and hydrophilic samples from granular soils. Previously, Dyvik and Olsen [65] reported on similar studies.

### 6.4. Triaxial Apparatus “In Situ”

The difficulties of sampling an intact soil for laboratory tests have led to the development of interesting field research proposals imitating a triaxial test.

New methods were simultaneously developed in Japan—dedicated mainly to rocky soils (patents: Ishibashi, Fukushima, Tani; e.g., [120,121]) and in France—dedicated to non-cohesive and cohesive soils, including weak soils (Laboratoire Central des Ponts et Chaussées patent according to Reiffsteck and Borel; [122,123,124]). The Japanese solutions are derived from core rock sampling methods and they are defined as the down-hole triaxial test for rock massifs [121]. So far, there have been a few publications in the scientific literature related to Japanese methods.

An unique device protected by Reiffsteck’s patent EP1226417B1/9913792 [123], filed by Laboratoire Central des Ponts et Chaussées in 1999, is another proposition dedicated for all soils. The ”in situ triaxial apparatus” was designed and made in cooperation between the “Center d’Étude et de Conception de Prototypes” in Rouen and LCPC represented by Reiffsteck and Borel [122]. In its concept, this device refers to the construction of a high-class, modernized triaxial apparatus and a self-boring pressuremeter. The apparatus itself, 1.55 m in length and 132 mm in diameter, is made of stainless steel. It weighs approximately 80 kg and consists of four main parts: (1) a pipe with the mounting system of sensors—850 mm in length; (2) upper cylinder with the loading system of sample—300 mm in length; (3) main part including: an appropriate sample, a membrane, the local sensors for measurement of strains and pore pressure—250 mm in length; (4) cutting edge—150 mm in length. The first publication related to the practical application of this device comes from 2008 [124].

The device is equipped with Hall and LVDT sensors for measuring axial and radial deformations even in the small strain range. Any stress paths can be performed thanks to the construction of the apparatus. The samples may be cylindrical or rectangular shape (possibility of determining the parameters under anisotropy conditions). They are cut from the soil below the surface at any depth and covered directly with a rubber membrane. Thanks to this solution within the stage of sample preparation avoided are the operations causing the most disturbances: transport, storage, and installation in the apparatus. This is why the in situ triaxial testing can be highly recommended in case of noncohesive and weak cohesive soils. The triaxial tests “in situ” can be performed almost continuously, one after the other, on successive depth levels. However, it is worth paying attention to the fact that the procedure of preparing the equipment for a test and test realization are very complicated. Perhaps, that is why the in situ triaxial apparatus has not been popularized.

## 7. Practical Guidelines Related to the Selection of Modern Displacement Measuring Systems

**Figure 22:**
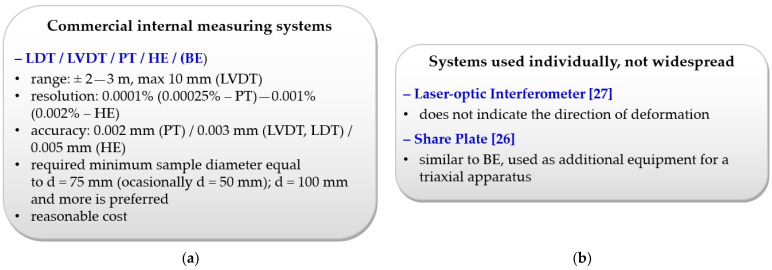

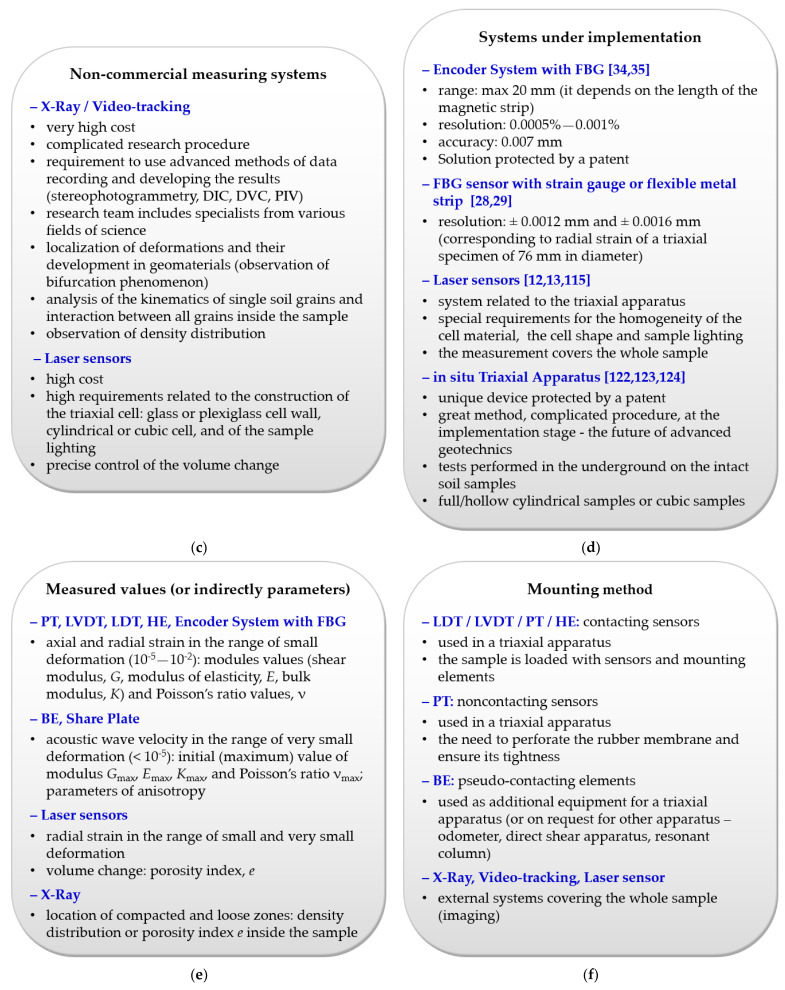

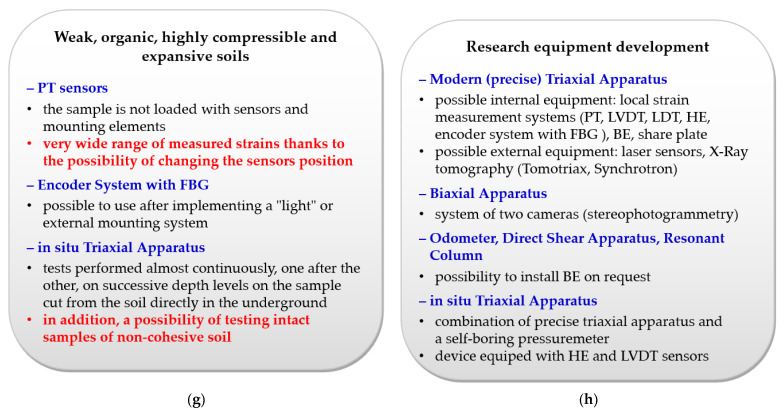
Guidelines for selecting the appropriate deformation measurement systems depending on: (**a**) Commercial internal measuring systems; (**b**) Systems used individually, not widespread; (**c**) Non-commercial measuring systems; (**d**) Systems under implementation; (**e**) Measured values (or indirectly parameters); (**f**) Mounting method; (**g**) Weak, organic, highly compressible, and expansive soils; (**h**) Research equipment development. (PT–Proximity Transducers, LVDT–linear variable differential transformer, LDT–local deformation transducer, HE–Hall effect gage, BE Bender elements, FBG–fiber Bragg grating).

This paragraph summarizes and complements the information presented in the preceding sections. Practical guidelines related to the selection of modern displacement measuring systems are presented in the form of “windows” (Figure 22). The selected criteria include, among others resolution, accuracy and range measurement, cost of the measurement devices, measurement complexity, system availability, purpose of measurement (measured values/parameters), type of soils, limitations and advantages, etc.

## 8. Final Remarks

The presented technologies and complex observation-measurement systems show the enormous progress that has been made in recent decades in the field of soil research. At the same time, they show the need for further improvement and optimizing the available equipment. One of the most important aspects is the necessity to adapt the test equipment to the evolving strain measurement systems.

The ongoing modernization of the triaxial apparatus is a very good example in the geotechnical laboratory. As is seen, thanks to such activities, most of the changes introduced in it are successfully transferred to other devices, e.g., odometer, direct shear apparatus, hollow cylinder, etc. Many of these modernized devices are offered for commercial sale by well-known companies of geotechnical equipment. Thanks to such innovations, the accurate measurement of deformation gives a realistic estimation of the deformation characteristics and reliable prediction of soil behavior under load, especially in the small and very small strain range, which most often correspond to the working conditions of the designed structures.

The presented research equipment review in the field of deformation measurement methods shows the newest possibilities of a modern geotechnical laboratory. A realistic understanding of the soil’s behavior “from the inside”, at the grain level, makes it an excellent tool for creating new soil models (or supplementing the existing ones), which can be later used in modern numerical modeling.

Usually, the use of the equipment discussed in this study, is costly and it serves mainly cognitive, but not commercial purposes. Traditional soil testing is sufficient for the majority of the designed objects. Of course, complicated structures (e.g., massive dams, skyscrapers, objects located on weak or seismic ground) require just such an advanced research-design approach. This is the future of geotechnics.

## Data Availability

The data presented in this study are available in the source materials included in the References.
